# Alveolar ridge keratosis - a retrospective clinicopathological study

**DOI:** 10.1186/1746-160X-9-12

**Published:** 2013-04-16

**Authors:** Lorenzo Bellato, Carla P Martinelli-Kläy, Celso R Martinelli, Tommaso Lombardi

**Affiliations:** 1 Laboratory of Oral and Maxillofacial Pathology, Division of Stomatology and Oral Surgery, School of Dental Medicine, Faculty of Medicine, 19 rue Barthélemy-Menn, 1205, Geneva, Switzerland; 2 Centre for Diagnosis and Treatment of Oral Diseases, Ribeirão Preto, SP, Brazil

**Keywords:** Alveolar ridge keratosis, Hyperkeratosis, Oral leukoplakia, Oral squamous cell carcinoma

## Abstract

**Background:**

Alveolar ridge keratosis (ARK) is a distinct, benign clinicopathological entity, characterized by a hyperkeratotic plaque or patch that occurs on the alveolar edentulous ridge or on the retromolar trigone, considered to be caused by chronic frictional trauma. The aim of this retrospective study is to present the clinicopathological features of 23 consecutive cases of ARK.

**Material and methods:**

The 23 biopsy samples of ARK were selected and pathological features were revised (keratosis, acanthosis, surface architecture, and inflammation). Factors such as the patient’s gender, age, anatomical location, tobacco and alcohol use were analyzed.

**Results:**

Sixteen out of the 23 cases studied were men and 7 women with a mean age of 55.05 (age ranged from 17 to 88 years). Thirteen cases had a history of tobacco habit, amongst whom, 4 also presented alcohol consumption. All the cases presented only unilateral lesions. Nineteen cases involved the retromolar trigone while 4 cases involved edentulous alveolar ridges. When observed microscopically, the lesions were mainly characterized by moderate to important hyperorthokeratosis. Inflammation was scanty or absent. In four of the cases, the presence of melanin pigment in the superficial corium or in the cytoplasm of macrophages was detected. None of the cases showed any features of dysplasia.

**Conclusion:**

Our results reveal that ARK is a benign lesion. However, the high prevalence of smokers amongst the patients might suggest that some potentially malignant disorders such as tobacco associated leukoplakia may clinically mimic ARK.

## Introduction

Alveolar ridge keratosis (ARK) is a lesion considered a distinct benign clinicopathological entity [1,2]. It has also been referred to as benign alveolar ridge keratosis (BARK) [3,4]. Clinically, ARK features a hyperkeratotic plaque or patch occurring on the alveolar edentulous ridge or on the retromolar trigone pad, believed to be caused by chronic frictional trauma (e.g. trauma during mastication). It can be compared to a callus on the skin [1] or may resemble the lichen simplex chronicus (LSC) of the skin [3], a lesion caused by chronic frictional injury. Histologically, the lesional epithelium shows hyperorthokeratosis, acanthosis and wedge-shaped hypergranulosis, and the surface can be slightly verruciform or corrugated. The inflammation ranges from absent to scanty. Although ARK is a benign entity, some potentially malignant disorders may clinically mimic it, such as idiopathic or tobacco associated leukoplakia [2].

Through this study, we present the clinicopathological features of 23 consecutive cases of ARK retrieved from our file.

## Material and method

The biopsy samples of ARK were selected from the archives of the Laboratory of Oral and Maxillofacial Pathology, Geneva, Switzerland. As retrospective study approval by the local institutional review board was not requested.

The 23 cases presented a clinical history of leukoplakia or white plaque lesion (keratosis) without erythema or ulceration limited to the retromolar trigone or the alveolar ridge region. In each case, the patient´s gender, age, anatomical location, tobacco and alcohol habit were evaluated. Pathological features were revised. These included the presence of keratosis, acanthosis, surface architecture, and inflammation.

## Results

**Figure 1:**
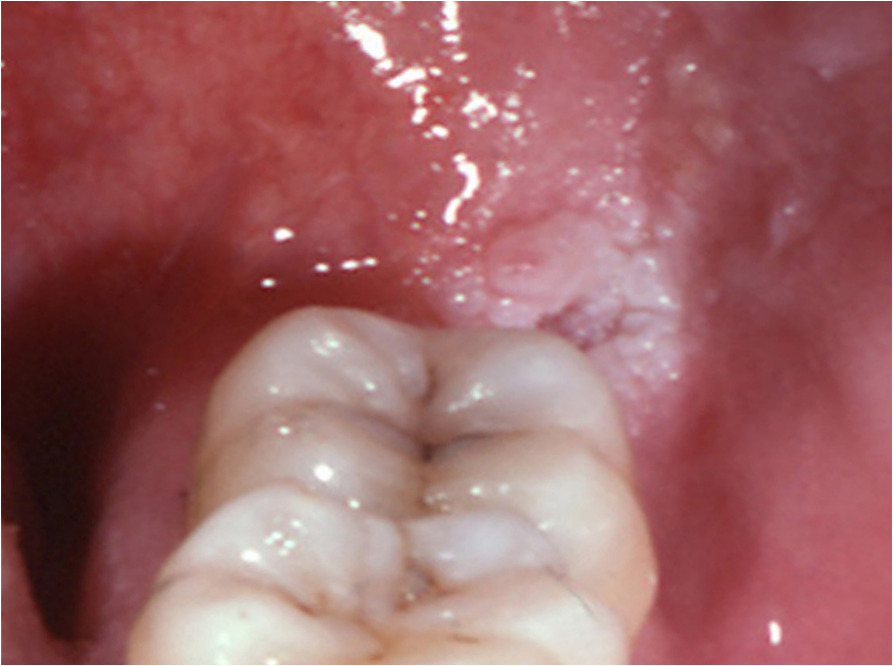
Clinical aspect of ARK: white lesion showing a corrugated surface on the retromolar trigone.

**Figure 2:**
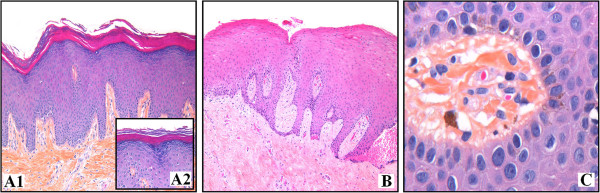
**Histological aspect of ARK: (A1) marked hyperorthokeratosis without dysplasia, discrete surface papillomatosis and mild acanthosis with elongated ridges, some of them anastomosed. (HES, × 10).** (**A2**) High magnification showing a wedge-shaped hypergranulosis. (HES, × 40). (**B**) Squamous epithelium presenting moderate to marked acanthosis and hyperparakeratosis with some anastomosed epithelial ridges and mild focal inflammation in the subjacent conjunctive tissue. (HES, × 10). (**C**) Melanin in the epithelium, free and in the cytoplasm of macrophages (HES, × 40).

Out of 23 cases, 16 were men and 7 were women (Table 1). The mean age was 55.05 (range 17–88 years); 19 cases (82.6%) occurred on the retromolar trigone (Figure 1), and 4 on edentulous alveolar ridges (17.4%). All the cases were unilateral lesions. A history of tobacco habit was present in 13 cases (56.5%), out of which 4 (17.4%) also presented an association with alcohol consumption. Only one case had no data available about tobacco (Table 1). Histologically, 22 out of 23 biopsies showed a moderate to important hyperorthokeratosis with wedge-shaped hypergranulosis (Figure 2A); only one case featured a hyperparakeratosis (Figure 2B). In addition, a variable acanthosis with some anastomosed epithelial ridges was observed (Figure 2A, B). None of the cases presented epithelial dysplasia. Inflammation was scanty or absent. In four cases were found either melanin pigment deposits or melanophages in the superficial corium (Figure 2C).

**Table 1:** Summary of clinical features of 23 alveolar ridge keratosis patients

**Clinical characteristic**	**N° of patients**
(n = 23)	(%)
Gender	
male	16 (69.6%)
female	7 (30.4%)
Location	
retromolar trigone	19 (82.6%)
edentulous ridge	4 (17.4%)
Tobacco habit	
yes	13 (56.5%)
no	9 (39.2%)
no information	1 (4.3%)
Alcohol habit	
yes	4 (17.4%)
no	16 (69.6%)
no information	3 (13%)

## Discussion

ARK is a benign traumatic lesion that has often been included in the leukoplakia category [1-4]. Woo and Nataraja have proposed the term BARK to emphasize the complete benign nature of ARK [3]. Chi et al. found dysplastic features in 10 out of 477 ARK cases that represented a true oral leukoplakia mimicking ARK [2].

In 2005, the definition of leukoplakia was discussed at the Oral Cancer and Precancer Workshop coordinated by the WHO Collaborating Center [5]. For this group, leukoplakia could be described as “white plaques of questionable risk, having excluded (other) known diseases or disorders that carry no increased risk for cancer”. In addition, its aetiology could be related either to tobacco or to areca nut use, or it might be idiopathic.

Leukoplakia may present some degree of dysplasia and also progress into verrucous carcinoma or squamous cell carcinoma (SCC). Studies have shown a frequency from 0.13 to 17.5 percent of malignant transformation of oral leukoplakia [6].

In the present study, the majority of ARK lesions showed hyperorthokeratosis, wedge-shaped hypergranulosis and variable acanthosis, with no dysplastic changes. In all the cases, inflammation was absent or scanty. Therefore, we agree that BARK related to trauma should be considered a distinct entity and consequently be excluded from epidemiological studies on leukoplakia. Nevertheless, other lesions may mimic ARK mainly when there is a history of tobacco habit [2]. In the present cases a high prevalence of smokers (56.5% of cases, see Table 1) was found, suggesting that ARK and tobacco associated leukoplakia (tobacco keratosis) might show the same clinical (and possibly even histological) features. In addition, 4 out of 13 cases that had a tobacco habit were also associated with alcohol consumption. Natarajan and Woo [4] also found a high prevalence of smokers and alcohol users in benign ARK: 13 out of 27 cases were smokers while 6 out of 27 cases were alcohol users. Furthermore, a study that used ARK cases as control samples showed 17% and 20% of positivity to HPV and p16^INK4a^, respectively. The p16^INK4a^ and HPV are entailed as a potential diagnostic marker for dysplasia and squamous proliferative lesions [7]. Recently, a paper described a series of 14 cases of frictional keratosis with similar features to those present in benign alveolar ridge keratoses, but exclusively located on the vestibular attached gingiva [8]. In these cases, only one had a history of pipe smoking, although he had quit smoking 10 years prior to the presence of the lesion. Yet, a history of a continuous trauma on the gingiva was reported and none of the 14 patients had alcohol consumption.

In some cases, the tobacco use can induce an oral mucosa pigmentation (smoker’s melanosis), in which the melanin can either be located in the basal cell layer of the epithelium or found free in the superficial corium or within macrophages [9]. In our study, 4 out of 13 cases that had a tobacco habit presented melanin pigment deposits in the superficial corium (Figure 2D). It is known that the increase of melanin in smokers is related to the stimulation of the melanocytic cells and that the degree of pigmentation is increased in heavy smokers [9,10].

**Figure 3:**
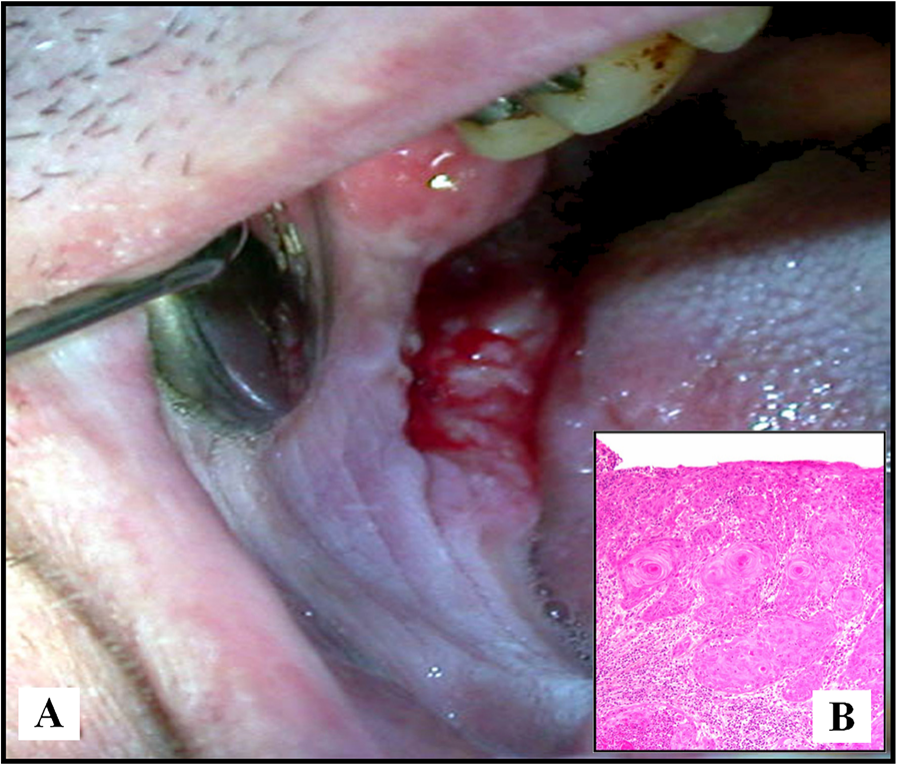
**(A) Clinical aspect of a SCC of the retromolar trigone.** (**B**) Histological aspect of the SCC (HES, × 10).

Tobacco has a strong association with (SCC) and heavy smokers present seven times more risk of developing cancer than nonsmokers. Its association with alcohol consumption is responsible for a greater proportion of oral cancers [1,11,12]. SCC can occur on the retromolar area (Figure 3) or edentulous alveolar ridge. From 225 OSCC cases studied, Kademani et al. [13] found 27 cases (12.4%) occurred on the retromolar trigone. Since early SCC can appear as a white lesion, it is also possible that some lesions (i.e. leukoplakia) may have the same clinical pattern of ARK.

ARK is a benign reactive lesion related to trauma that is difficult in, some cases, to differentiate from a tobacco lesion especially if located on the retromolar area. It is, therefore, possible that tobacco associated leukoplakia (tobacco keratosis) might be considered as ARK. In patients with an ARK it is important to investigate the occurrence of tobacco use and alcohol consumption and to perform a biopsy in order to confirm the diagnosis. We concur with the opinion that benign ARK should not be included in epidemiological studies on leukoplakia.

## Competing interests

The authors declare that they have no competing interests.

## Authors’ contributions

LB and CPMK collected the data. TL and CPMK performed histopathological examination. CPMK and TL analysed and interpreted the data. The concept of the paper was devised by CPMK and TL. LB, CPMK, CRM and TL wrote the manuscript. All authors read and approved the final manuscript.
